# Rural Indonesia women’s traditional beliefs about antenatal care

**DOI:** 10.1186/1756-0500-5-589

**Published:** 2012-10-29

**Authors:** Yenita Agus, Shigeko Horiuchi, Sarah E Porter

**Affiliations:** 1 St. Luke’s College of Nursing, 10-1 Akashi-cho Chuo-ku, Tokyo, 104-0044, Japan; 2 Oregon Health & Science University Portland, Oregon, USA; 3 Syarif Hidayatullah State Islamic University, School of Medicine and Health Science, Jl. Kertamukti Pisangan Ciputat, Jakarta, Indonesia; 4 St. Luke’s Birth Clinic, 1-24 Akashi- cho, Chuo-ku, Tokyo, 104-0044, Japan

**Keywords:** Pregnancy complications, Rural women, Traditional beliefs, Traditional births attendant

## Abstract

**Background:**

The Indonesia Maternal Mortality Rate (MMR) of 420/100.00 live births remains among the highest in East Asia while coverage of births assisted by skilled providers is still low. Traditional beliefs have been a key factor associated with the choice between midwives or traditional birth attendants (TBA) and the low number of antenatal care visits in rural West Sumatra.

**Methods:**

We conducted three focus groups with 16 women from rural West Java to describe their perception regarding issues related to traditional beliefs. Focus group discussions provided data for the content analysis.

**Results:**

The majority of the 16 women interviewed was from Village Dago, West Java and had only an elementary school education. Their ages ranged from 19 to 40 years. Most were multiparous housewives with an income of IDR 918.750 per month, which was lower than the monthly income in West Java (IDR. 1.172.060). Emerging from the focus group discussion were four main themes regarding their pregnancy and traditional beliefs: 1) pregnancy was a normal cycle in women’s life (pregnancy is a natural phenomena, not a sickness; no recognition of danger signs during pregnancy and death of baby or mother during pregnancy was brought about by God’s will); 2) women followed the traditional beliefs (positive motivation to follow the traditional beliefs and fear of not following the traditional beliefs); 3) relying on TBA called *paraji* rather than midwife (*parajis* are kind, tolerant and patient and have more experience than midwives; more accessibility than midwives and encouragement of natural birth) and 4) midwives are more secure than *paraji*; (they use a medical standard of care).

**Conclusions:**

Women’s beliefs grounded in religion and tradition permeated the village culture making it difficult to counter their long held health practices with practices based on recent advances in health care. Use of TBA in this village was still dominant and women believed that following traditional beliefs led to a healthy pregnancy therefore, they also followed all relatives’ suggestions. Understanding the complexities of local culture is the first step to improving women’s awareness of how to preserve their pregnancy and prevent complications.

## Background

Improving maternal health is one of the eight Millennium Development Goals adopted by the international health community in 2000 [1]. The Indonesia Maternal Mortality Rate (MMR) remains among the highest in East Asia with 420 deaths per 100,000 live births while, 10,000 women continue to die of childbirth-related causes every year [2,3]. The WHO report [2] noted that coverage of births assisted by skilled providers is still low especially in rural areas, where traditional births attendants (TBAs) assisted more than 50% of deliveries. In other words TBAs who have minimal training attend the majority of births [4].

The high cost of medical expenses, poor quality of emergency obstetric care and an inadequate number of health specialists to manage complications continue to discourage women from seeking care when needed. In addition, shortage and maldistribution of health providers are problems requiring attention [3,4]. Despite the lack of qualified health care providers, a previous study [5] found that traditional beliefs were the greatest contributing factor influencing the preference for TBAs. It was also identified that some of the women especially in rural areas agreed that following the traditional belief was necessary. The UNFPA report found that millions of women were affected by cultural traditions that had implications for their well-being, including the denial of modern medicine during childbirth [6].

Culture is how individuals learn the values, beliefs, norms and way of life that influence their thinking, decisions and actions in certain ways [7]. It is presumed that this discrimination has been woven into the traditional cultural. Family as a unit of culture is therefore an influencing factor for women in following the traditional beliefs and also encouraging women to seeking care during pregnancy [5,8]. For example, most women in Bali believed that trusting family advice is necessary [9].

Women’s preference for TBAs was more that just a tradition, because TBA’s provided the patient with kindness and caring; they had skills plus respect for the local customs which were highly valued [10,11]. The TBA understood the local customs. The health care professionals, usually coming from or being educated from a different perspective often failed to understand the significance of the traditional practices and had little knowledge about the traditional beliefs [12].

### Statement of the problem

While there are multiple interacting factors affecting birth outcomes in Indonesia there remain areas where women could chose to access trained midwives as opposed to the TBA. However, many women especially in rural areas still prefer to go to TBAs for assistance during pregnancy. They believe that TBA services are part of the traditional beliefs during pregnancy. This situation is predicted as one of the factors contributing the low number of ANC visit in Indonesia.

In-depth understanding of antenatal care in relationship to traditional beliefs requires investigation using in depth interviews. Health care providers need to know the traditional beliefs of the women, families and communities for whom they provide care; through women’s own discussion about their health problems and ANC services their perceptions will become more easily understood. This study focuses on the factors regarding preferences for TBAs or midwives and the role that traditional beliefs might have in their decision.

### Purpose

The aim of the study was to describe women’s perception regarding issues related to traditional beliefs during their pregnancy in rural area of Indonesia. The study explored women’s perception regarding traditional beliefs, including their reasons for using or not using ANC services during their pregnancy. Women’s perceptions were interpreted within the context of traditional beliefs related to views about pregnancy and health care.

## Methods

### Design

This study used a qualitative exploratory cross-section design because little was known about women’s perceptions of antenatal care and traditional beliefs.

### Setting and sample

#### Characteristics of the setting

The data were collected in the rural area near Bogor in West Java, a province of Indonesia. The capital of the province is Bandung. According to the Indonesian Demographic Health Survey [13] 18% Indonesia women graduated from secondary school whereas 22% of men completed secondary school.

#### Sample and recruitment

The researcher chose the geographical area based on purposive sampling. Areas selected were based on health data of the women such as the number of women who delivered with TBAs and the distance to public health services. After the areas were selected village midwives, head of the neighborhood and village health volunteers recruited participants based on the inclusion criteria: 1) married women; 2) expecting birth or had experienced birth (TBAs or Midwives); 3) lived in the village and 4) able to communicate in the Indonesia language. Recruitment by the village midwives and village health volunteers (Kader) continued until there were enough participants for 3 groups with 5–6 for each group. The researcher collected data from April to May 2011.

### Data analysis

Data were collected from the focus groups led by the researcher and a trained research assistant using semi-structure questionnaires guiding the focus group discussion. Content analysis to determine who said what followed Ezzy’s [14] five-step process: (1) Early summaries of the interview were made immediately after transcribing and reading of the interview to obtain a sense of the content; (2) All interviews were coded manually and a large number of categories were outlined and grouped together under higher order headings; (3) Similar headings were removed and categories were generated to reflect the study aims; (4) Trustworthiness of data analysis was addressed by asking a colleague to generate the theme list independently; (5) Every transcript was coded based on the list of themes and then moved to the theme where it belonged.

### Ethical consideration

The Ethics Review Board in St. Luke’s College of Nursing Tokyo, Japan approved the study. The district health office (*dinas kesehatan kabupaten*) and community health center (*puskesmas*) in West Java, Indonesia also approved the study. This study was conducted based on the protection of human subject’s provisions from the Declaration of Helsinki including anonymity and confidentiality. The objective and significance of this study and the procedures and ethical considerations were explained to the health care staff to gain their cooperation and assistance in this study. After approval of both the ethics committee and district health office the researcher explained the study to the village midwives and village health volunteers (*kader*). The researcher explained the study emphasizing participant’s rights to withdraw at any time without penalty. Informed consent was received from each participant. The collected data will be kept in a safe place for at least three years. After publication, all data will be shredded.

## Results

### General characteristic of the women

As was found throughout Indonesia, most women in this village were Muslim. Of the 16 women interviewed the majority were from Village Dago, West Java. Only six of the 16 women delivered with a midwife and they chose to deliver at home. Yet, all the women went to the *paraji* for a check-up especially at the fourth and seventh months of pregnancy. In this area the *paraji* was an important tradition during pregnancy. At four months and seven months of pregnancy, the *paraji* provided a massage for pregnancy. In essence, the women believed that in order to maintain their infant’s health, they needed to take a good care of themselves and follow the cultural rules.

There was a range of issues that influenced women views of the traditional belief during pregnancy. Four global themes emerged: (1) pregnancy is a normal cycle in women’s lives; (2) women follow traditional beliefs; (3) follow traditional beliefs relying on *paraji* rather than midwife and (4) midwives are more secure than *paraji*.

### Pregnancy is a normal cycle in women’s life

#### Pregnancy is natural phenomena not sickness

Most respondents from this village said pregnancy was a normal experience, which should not be regarded as a disease or pathological state. The women also considered pregnancy to be a normal period in their life cycle. They believed that pregnancy would bring them only happiness, even though they did not have any special desire for babies.

One woman said “*I think pregnancy is a normal process so you do not need to think bad thoughts about it*”. Another woman mentioned, “*Delivery also is a normal process*, *even though nobody helps you and the baby will come out itself*”.

In this case women reported that view regardless of the feasibility that a complication could occur, because they did not think something would happen to them during the process. Therefore danger signs were not neither anticipated or looked for nor even recognized.

#### Not recognizing danger signs during pregnancy

Almost all of the women failed to identify the danger signs of pregnancy. Many of the women did not think a headache was a problem during pregnancy. A respondent said: “*I think a headache during pregnancy is a normal sign*, *there is no need to worry about that*”. Most women had experienced a headache during their pregnancy. One woman said, “*I think a headache will come if you eat many foods containing acid like orange or lemon*. *But there is no danger and it did not cause trouble with their pregnancy*”. Many of women identified that those symptoms are not necessarily related to pregnancy. They agreed, “*There are many causes of headache*, *if they are late to take breakfast*, *or are too tired*, *surely a headache will come*. *However*, *a headache is not about your pregnancy*, *so you just take a rest and eat regularly and it will be going by itself*”. However, another woman believed when they had some problems, for example headache, they would be better off if they went to the *paraji* and received a remedy. One woman said, “The *paraji gave me Jampe* (*sprayed water*), *I feel better after paraji*’*s therapies*”. She said, ”*jampe was used for exorcism*”.

Not all women believed that self-care or care by the *paraji* was the correct action. On the contrary, a few women said headache is problem during pregnancy and thus sought a check up by midwives, doctor or went to the hospital when they had a headache. A few women agreed, “*Headache is not a normal sign*, *because headache is one of the signs of hypertension*. *So*, *we need to get a check*-*up regularly with midwives*”. Another woman said, “*If headaches are frequent during your pregnancy then that is a great risk and also a risk for your baby*”. Another woman said,” *it was a very big problem and I delivered by caesarean operation*, *so we need to consider going to midwives if we had headache during pregnancy*”. The prevailing belief however was in the normalcy of pregnancy with whatever risks ameliorated by traditional diet and behaviors.

#### Infant and mother’s death during pregnancy is brought about by God’s will

During the focus group the women mentioned that only a few women and babies died during pregnancy; even if it happens, it is because God wanted it this way. One woman said, “A woman was delivered by a *paraji* and her baby died after delivery. The women said, “*Her baby died because of umbilical infection and she knew it was what God wanted and she only surrendered and believed this is fate*. *So she prays to God to keep us safe*. “*That is the only thing we will do*.”

### Women follow the traditional beliefs

#### Positive motivation to follow the traditional beliefs

During the period of pregnancy and childbirth women changed a variety of health–related behaviors including types of food consumed and their activities. There were specific culturally defined prohibitions on eating certain foods during pregnancy and childbirth though not all women followed this cultural rule. Women believed that in order to make themselves healthier they needed to take good of them, as well as follow the traditional beliefs and pray to God to make them safe.

Generally, the women’s beliefs about what types of food that should and should not be eaten during pregnancy and childbirth were common within the wider population. A woman said, “*Drinking soda* (*coca cola*, *sprite*) *was not good for pregnancy*; *it will make contractions and your baby will be damaged*. Another woman stated “*vegetables are better after delivery than meat or fish*, *because the fish makes the breast milk smell and the taste is bad for the baby and people surrounding them*”. Women followed the cultural rules of eating to make them selves healthier.

#### Afraid to not follow the traditional beliefs

Even though many women had a check-up with a health care provider at the Integrated Health Service Posts (*Pos Pelayanan Terpadu* (*Posyandu*), and most of them received information from a midwife about health care during pregnancy and delivery the women said that following the traditions and advice from their grandmother and mother was more important. They followed traditions even though they did not understand why and they believed that if they disobeyed the ritual it would have a negative impact on them. For example “*they should avoid going outside at night without jimat* (protection such as scissors, garlic, and knife)”. Almost all traditional beliefs and practices identified by women were directed toward the consequences of not adhering to the practices. For example one woman said that she went out at nighttime without the *jimat*, and after that she felt she had a devil in her home. One woman said “*I felt uncomfortable if I disobeyed these rituals*, *this is our tradition*, *and we will follow all of the traditions even if we could not understand why*. *It is true*. *If we did not follow the rituals*, *after that something will happen to you*”. Most of women believed that some traditional rituals should be obeyed. They believed all the rituals had a benefit and felt uncomfortable if they did not follow it.

### Relying on *paraji* rather than midwife

#### *Parajis* are kind, tolerant and patient and have more experience than midwives

The services of TBA (*p*a*raji*) were commonly used in Dago Village during the antenatal and childbirth periods. Some women said, in seeking safety and comfort they had a strong belief that the *paraji* would come help them and would be with them whenever they wanted them. Furthermore *paraji*’*s* were more patient; they gave them support and were helpful people. *One woman said*, “*I delivered with midwives at home but paraji also was here to help me*, *and usually after delivery paraji did a kind of massage to restore my body like it was before*. *Paraji*’*s are also responsible for taking care of the cord and bathing the newborn*. Women reported that the *paraji* lived in their community and they have known the *paraji* since they were young and had a good relationship. On the contrary midwives were only available during the process and could not help them as much like the *paraji* did.

#### More accessible than midwives

In this area where one village midwife was available, traditional births attendants were more capable of reaching the community. The community also perceived their role to be as important as the *Ustadz* (religious leader). Some women preferred using the services of the *paraji* to health care professionals such as the village midwife. One woman said, “*I do not need much money to pay the paraji and the paraji did not push us to pay and we will pay when we have money or something to give them*.” Some said, “*I needed to prepare more money to use midwives services*, *on the other hand using the paraji service we do not need transport because the paraji lives within our community*”. Regarding transportation they said, “*If you delivered at the health centre it was too far away to be reached and also it*’*s hard because you need to use a car or motorcycle*”. However *parajis are part of the community* and easily accessible. The common mode of transportation in this village was by motorcycle or walking. In some areas their homesteads were very far away from the regular routes of the commuter motor coach. At night it became difficult to borrow such a car. This was an undesirable situation as it would also be expensive. The women thought money was one barrier for them in seeking health facilities.

#### Encouragement of natural birth

Some of women mentioned that the *paraji* is always besides them during the process, kind and patient and always waiting until the baby comes out naturally. They said, “*Midwives performed an episiotomy to make the baby comes out faster*; *sometimes midwives did not have time*, *so they did another intervention*.” Only a few of the women had experienced birth at the hospital. They believed birth at home was a normal process. One woman said “*I preferred delivery with a midwife*; *even when delivering with a midwife I did not want to deliver at the hospital or midwife*’*s clinic*. *It makes me worried*. *The people in this village would think something bad happened with me*. *I did not want to make the people thinking badly about me*.” Thus, they only sought midwives if they had some problem with their delivery.

### Midwives more secure rather than *paraji*

#### Using a medical standard

However, a few of women preferred using the services of health professionals. One woman said, “*The paraji waited a long time until the baby come out*; *it made me worried whether she could handle the situation*. *She waited and did not think whether I still had energy or not*. *But*, *midwives would give us an intervention to restore our energy through intravenous injection if the delivery was longer than usual*.” Another woman said, “*Midwives give the appropriate care during the process and made us feel safe*”. One woman said, “*The paraji was not so secure*; *sometime they used a strange method and did not use a glove*”.

In summary, the practices of *parajis* were important in this community. Women seemed to favor the traditional belief option for the following reasons; the *paraji* was easily available, affordable and accessible to the women. The beliefs about health during pregnancy followed traditional beliefs and they followed all relatives’ suggestions. They believed God would determine what would happen in their life, so they kept praying and believed that fate was important.

## Discussion

The findings of this study have shown that the ultimate focus of all decisions made by the women who lived in the Village Dago related to traditional beliefs. What they described and what could be observed was a range of different behaviors dependent on their interpretations of concrete life situations. Influencing factors on beliefs was that pregnancy is a normal cycle in women’s life, women follow traditional beliefs, women rely on the *paraji* rather than midwife and a few of women thought midwives were more secure rather than the *paraji*. The in-depth interviews using focus group discussion have shown that the women in this community had various beliefs about how to manage pregnancy.

### Pregnancy is a natural life event

Women believed that pregnancy was a normal cycle in a woman’s life, which did not need special care during the antenatal and childbirth periods; home delivery was more acceptable than traveling to a clinic or hospital. Studies from a variety of cultures in less developed countries have also identified similar beliefs. Similarly, a qualitative study in Bangladesh and Australia [15,16] reported pregnancy identification and its subsequent care was seen as a normal event and was not considered special consideration unless significant complications arose. In addition, an ethnography study by Janson, [17] in Ghana found that some pregnant women in a Ghanaian Village also described childbirth as being something natural and not an illness, which does imply that the pregnant woman can deliver at home.

Additionally some symptoms during pregnancy for example, headache, were considered a normal sign. Agus et al. [5] found that only 62% of women correctly answered the item: ‘headache is a normal sign during pregnancy’. If they needed something for other symptoms including headaches they believed going to the *paraji* and receiving a remedy would make them feel better. More over, the practice for taking traditional herbal remedies during pregnancy had been passed down for many generations and there were no associated side effects in Indonesia [9].

Although some of these beliefs were culturally understandable, women proceeded on the belief that if they followed the traditional beliefs, practices and advice of family and the *paraji* then they had done all that they could and the remainder of the outcome was in God’s hands. In this village, the women believed that babies die during the process of delivery because of God wanted that way. Belief in God and acceptance of all of God decision was absolute; religion also had an impact on women’s perception of how to maintain and preserve their pregnancy. Similarly, a study in Zambia found that illness during pregnancy was explained by culturally accepted causes. They believed a physically and spiritually weak state made one more susceptible to illness, sickness, witches and evil forces in the environment and they accepted it as God’s will [18]. Mathole et al. [19] confirmed that Zimbabwe women believed that the first three months of pregnancy were crucial and sensitive; it was believed that a pregnant women and the pregnancy are vulnerable to witchcraft during the early period of pregnancy.

Although pregnancy was considered a normal event in women’s live and God is one who gives the decision, following the health care provider information is one part of their effort to preserve their pregnancy. From the medical perspective women can get into trouble during the delivery without her knowledge, because most obstetric complications occur around the delivery period and often cannot be predicted. Therefore, they need special attention for their pregnancy to avoid complications, which can be addressed with certain preventive measures by a professional health care provider. *More over*, *the health provider should understand well the women*’*s belief in order to simplify giving the health information*.

### Women tend to keep and obey the traditional beliefs

This study found women followed the traditional beliefs. They believed that following the traditional rituals and praying to God would make them become healthier. As a result, one area talked about was what types of food should and should not be eaten during pregnancy and childbirth.

Other cultures have also developed food taboos for pregnant women. Chinese women in Hong Kong found that dietary taboos during pregnancy were: eating shrimp because it will cause skin allergies, rabbit meat, which will cause cleft plates in newborn babies and beef which will also affect fetal health [20]. Fear of not following the traditional beliefs was common in this village; they stated they felt uncomfortable if they disobeyed these rituals. A study in Limpopo South Africa found that to prevent malformation of the fetus, which could be inflicted by jealous people, pregnant women needed to be supported physically and spiritually and also needed be strengthened with herbs [21]. An interesting perspective presented by Camacho, [22] stated that among indigenous women, taboos and permissions during the reproductive cycles were strongly linked to the sacred dimension of nature and spaces were related to the knowledge, practices and rituals.

Empowerment of women is a key strategy in strengthening women’s capacities to make healthy decisions for themselves and their families. The empowerment of women is one of outcomes of women centered care (WCC) [23]. Women who had an opportunity to make a decision about choice of care, they felt their views were acknowledged [24]. In addition women who had a strong desire to make decisions were more likely to give birth at a health facility than women with little decision making power [25]. Promoting the exchange of knowledge is a strategy to expand the learning process among women and health care providers. This will help strengthen the effectiveness of the responses of health care providers for the specific needs of women. Empowering women’s activities should be woven into health education strategies that promote cultural changes that can benefit women and families.

### Who were more affordable health providers the *Parajis* or midwives?

We found a main factor determining the type of health care provider for some women in this village was economical. They selected the *paraji* because she was more financially feasible. Another reason found was that the *paraji* was felt to be more kind and patient and more capable of reaching the community. Similarly Titaley’s study [11], found the reason for using the services of the *paraji* were economic, trust, tradition and easy access. In South Africa women agreed that the *parajis* were knowledgeable in what they did and had skills for pregnancy care [21].

Access between the community and health care center and health care provider also emerged as an issue. For some, home delivery was considered more convenient for some women because they thought they had good health. The home environment was more comfortable than the hospital, and they received support from family members [21]. Women trusted the *paraji* because they knew them, as they were from the same community and some of their family members were also using the same services during their pregnancy.

Another finding was that a few women in this village stated midwives were more secure rather than the *paraji*. Wulandari’s [9] qualitative study using in-depth interviews in Bali Indonesia found that women stated they only go to midwives and never believed the *paraji*. On the contrary, women in South Africa stated that they were sometimes reluctant to visit clinics due to the attitude of the nurses [21].

Women who preferred a *paraji* and those who preferred a midwife both agreed that the *paraji* was still needed to maintain their pregnancy; they had a strong belief that at the seventh month of their first pregnancy they should go to *a paraji* to receive a special massage. Therefore, the roles of village midwives and *parajis* were perceived as vital, particularly in that rural area.

Midwives as health care providers provided comprehensive care during pregnancy. However to increase women’s use of midwifery services requires the adoption of some strategies because village midwives were perceived as unacceptable to women and their family. Village midwives need to consider the traditional understandings and beliefs and find a way to be more accepted within the community. One strategy must be to establish a relationship of trust within the community. Trust is an important factor for increasing the use of health provider services. With increasing trust the midwives should be able to conduct more effective health education including important risk factors and actions to take especially if women are primarily using the services of the *paraji*. Moreover, increasing the *paraji*s knowledge and skill base including appropriate referrals, cultivating a referral system and facilitating the acquisition of health insurance would address part of the motivation to use only the *paraji*.

### Limitation and further research

There were several limitations of this study: First, was the small number of women participating in the focus group therefore, the results are not transferable to all Indonesia women. Second was possible bias in subject selection related to interpersonal relationships within the village. Third was the data collection method. Focus groups, while useful for generating rich discussion, have the drawback of not always capturing varieties of opinion because of social conforming especially in some cultural settings. Even so, this study found several noteworthy issues about women’s understanding and experiences about pregnancy.

Developing a trusting relationship between midwives and the community is perceived as vital; traditional beliefs should be discussed among midwives, policy makers and planners to increase their understanding as to how to develop strategies for increasing midwives’ services. Improving women’s knowledge regarding danger signs during pregnancy is a top priority for women. To make them aware about danger signs, improving knowledge is one strategy to avoid complications during the process. A randomized controlled trial study by Liu [26] found that health education for the intervention group was successful in improving pregnant women’s health knowledge and some postpartum practices. Future research should focus on describing the beliefs and practices of more women in the area and on the development of strategies for how midwives can improve health education and awareness about women needs. Empowerment of women is one possible strategy for those sets of problems.

## Conclusions

In this study the most dominant provider chosen by women in the village was the *paraji*. The reasons for this choice were: services of the *paraji* were kind, inexpensive, interwoven in the community and easy to access. Women believed that because pregnancy was a normal life event, they did not need special consideration during pregnancy. By following their relative’s suggestions and trusting in God’s will, women thought they would have nothing to fear. These were the important beliefs for maintaining a healthy pregnancy. These were the traditional beliefs. Therefore to be accepted in the community it is vital for the midwife to understand the community beliefs and to develop a trusting relationship between health care provider and community.

## Competing interests

The authors declare that they have no competing interests.

## Authors’ contributions

YA and SH were responsible for the study conception and design and the drafting of the manuscript. YA conducted the focus groups and the data collection undertook the data analysis. YA, SH and SP performed the interpretation of the data and editing the paper. All authors read and approved the final manuscript for submission.
